# From classic to innovative: The continuation and modern application of calcium phosphate co-precipitation method in mRNA tumor delivery

**DOI:** 10.1016/j.omtn.2024.102384

**Published:** 2024-11-29

**Authors:** Jiayu Zhang, Jun Chen

**Affiliations:** 1 CAS Key Laboratory for Biomedical Effects of Nanomaterials and Nanosafety, Multi-disciplinary Research Division, Institute of High Energy Physics and University of Chinese Academy of Sciences (UCAS), Chinese Academy of Sciences (CAS), Beijing 100049, P.R. China

## Main text

In their recent study, Ohta et al. have introduced a pioneering method for delivering mRNA into tumors using an innovative approach that combines phosphate-buffered saline (PBS) and calcium ions to form nanoparticles with mRNA.^1^ This technique allows for the successful transfection of several tumors through intratumoral injection. The authors utilized circular dichroism spectroscopy and transmission electron microscopy to observe the conformation of mRNA altered in PBS, with calcium ions facilitating the formation of mRNA nanoparticles within this medium. By employing this method, they transfected mRNA encoding OX40-ligand, interleukin 36γ, and interleukin 23, resulting in suppressed tumor growth.

The narrative surrounding this research is reminiscent of earlier work; after all, the calcium phosphate co-precipitation method has been a ubiquitous transfection technique since the 1970s, as articulated by Graham and van der Eb in 1973.^2^ The principle involves mixing DNA with calcium chloride in a phosphate solution to create a calcium phosphate-DNA co-precipitate that is then dispersed onto cultured cells. Calcium phosphate facilitates the binding of DNA to cell surfaces, ultimately allowing DNA to enter cells via endocytosis. This classic methodology continues to find its place in molecular cloning laboratory protocols around the globe. Interestingly, the method employed by the authors is very much aligned with the traditional calcium phosphate transfection method, diverging only in that they are transfecting more unstable mRNA.

However, the calcium phosphate co-precipitation transfection method is notably sensitive to slight variations in pH, temperature, and buffer concentration, raising concerns regarding its cytotoxicity, particularly to primary cell cultures.^3^ Moreover, this technique is less suited for the systemic transfer of nucleic acids *in vivo* compared to other chemical transfection methods like lipid-mediated transfection, which might account for the authors’ choice of intratumoral injection.

The timing of this report is particularly fortuitous, as the COVID-19 pandemic led to the fastest approval of mRNA vaccines in history, taking a mere 10 months from concept to market.^4^ This remarkable achievement can be attributed to the groundwork laid by earlier research into lipid nanoparticle (LNP) delivery technology. Although LNPs have proven successful, they face challenges such as patent issues, allergic reactions, and high costs. Thus, the authors’ method represents a significant exploration into addressing the cost of nucleic acid delivery systems.

In conclusion, the development of new delivery systems is paramount for driving forward innovation within the realm of gene delivery. The work by Ohta et al. not only builds upon a rich history of transfection methods but also carves a path toward more accessible and potentially less expensive options for mRNA delivery—an essential step in the ongoing quest to harness the full therapeutic potential of mRNA technologies in oncology and beyond.

## Acknowledgments

We are grateful to the 10.13039/501100021171Guangdong Basic and Applied Basic Research Foundation (2022A1515140073), the 10.13039/501100001809National Natural Science Foundation of China (21574136), and the Youth Innovation Promotion Association Program of CAS (2015008).

## Author contributions

J.Z. prepared the original draft, and J.C. reviewed and edited the manuscript.

## Declaration of interests

The authors declare that they have no known competing financial interests or personal relationships that could have appeared to influence the work reported in this paper.
